# *IDH1/IDH2* but Not *TP53* Mutations Predict Prognosis in Bulgarian Glioblastoma Patients

**DOI:** 10.1155/2014/654727

**Published:** 2014-04-24

**Authors:** Gergana Stancheva, Teodora Goranova, Maria Laleva, Margarita Kamenova, Atanaska Mitkova, Nikolay Velinov, George Poptodorov, Vanio Mitev, Radka Kaneva, Nikolay Gabrovsky

**Affiliations:** ^1^Molecular Medicine Center, Medical University of Sofia, 2 Zdrave Street, 1431 Sofia, Bulgaria; ^2^Department of Medical Chemistry and Biochemistry, Medical Faculty, Medical University of Sofia, 2 Zdrave Street, 1431 Sofia, Bulgaria; ^3^Department of Neurosurgery, University Multiprofile Hospital for Active Treatment and Emergency Medicine “N.I.Pirogov”, 21 Totleben Boulevard, 1606 Sofia, Bulgaria; ^4^Department of Pathology, University Multiprofile Hospital for Active Treatment and Emergency Medicine “N.I.Pirogov”, 21 Totleben Boulevard, 1606 Sofia, Bulgaria

## Abstract

Mutations in genes encoding isocitrate dehydrogenase isoforms 1 (*IDH1*) and 2 (*IDH2*) have been associated with good prognosis for patients with brain neoplasias and have been commonly found together with mutated *TP53* gene. To determine the prevalence of *IDH1*, *IDH2*, and *TP53* mutations and their impact on overall survival 106 glioblastoma patients were analysed. *IDH1* mutations were detected in 13 and *IDH2* mutation in one patient. Two homozygous samples with R132H mutation in *IDH1* gene and a novel aberration K129R in *IDH2* gene were found. Sixty-four percent of *IDH1/IDH2* mutated tumours harboured also a mutation in *TP53* gene. Genetic aberrations in *TP53* were present in 37 patients. Statistical analysis of the impact of the studied factors on the overall survival showed that the mutations in *IDH1/IDH2*, but not the ones in *TP53*, were associated with longer survival. Also, the impact of age on prognosis was confirmed. This is the first comprehensive study on glioblastomas in Bulgaria. Our results suggest that *IDH1/IDH2* but not *TP53* mutations together with other prognostic factors such as age might be applied in clinical practice for prediction of outcome in patients with glioblastomas.

## 1. Introduction

The most common primary brain tumours are gliomas. The worldwide incidence of the most malignant form, glioblastoma multiforme (GBM, WHO grade IV glioma), is 2-3/100000 people per year [1] with a median survival of 6–12 months [2, 3]. In spite of the existing classification, glioma subgroups are not homogeneous in terms of survival [1]. Several prognostic factors have been proposed: age, extent of resection, and KPS, and also some molecular markers [4–6].

A study has revealed somatic mutations in a gene encoding isocitrate dehydrogenase 1 (*IDH1*) in 12% of GBMs [7]. Isocitrate dehydrogenases (IDHs) belong to a family of enzymes catalyzing oxidative decarboxylation of isocitrate to *α*-ketoglutarate. The human genes* IDH1*,* IDH2*,* IDH3A*,* IDH3B*, and* IDH3G* encode three IDH-enzymes: NADP+-dependent IDH1 localized within cytoplasm and peroxisomes, mitochondrial NADP+-dependent IDH2, and NAD+-dependent IDH3, respectively [8–10]. Mutations have been found not only in* IDH1* and* IDH2*, predominantly in gliomas, but also in leukemia samples and very rarely in other cancers [11, 12]. Three amino acid residues, Arg132, Arg109, and Arg100, participate in the formation of the catalytic centre involved in isocitrate binding. Almost all published mutations in* IDH1* affect Arg132. Genetic aberrations in* IDH1* have been reported in 50–80% of gliomas WHO grade II to IV [13–17]. Only primary GBM shows low frequency of* IDH1* mutations: 5% [17]. Approximately 90% of the R132 mutations are of the R132H type, followed by R132C changes in 4% and R132S and R132G in about 1.5% each, and very rarely the R132L mutation [10, 16, 17]. The* IDH2* aberration in Arg172 residue is an analogue of the one in Arg132 of* IDH1*. Mutations in the* IDH2* gene have been detected in up to 3% of glial tumours WHO grades II and III but not in GBM [17, 18]. Aberrations in both* IDH1* and* IDH2* genes have been associated with better prognosis in glioma patients of various grades [19, 20].

In a recent large scale study over 80% of the gliomas with* IDH1 *aberrations carried* TP53* mutations and/or 1p/19q loss (mainly in oligodendrogliomas) [17]. The tumour-suppressor gene* TP53* encodes a protein p53 implicated in the pathogenesis of many cancers.* TP53 *mutations have been reported not only in about 30% of gliomas [21, 22], mainly low-grade ones, but also in secondary glioblastomas (65% versus 28% in primary GBM) [23]. In secondary GBM over 50% of all mutations in* TP53* have been observed in codons 248 and 273 whereas in primary glioblastomas only 17% occur there. While most* TP53* aberrations resulted in decreased apoptosis in response to DNA damage, thus enabling tumour growth and influencing negatively patient's overall survival, those in codons 248 and 273 have been associated with better survival for patients [7, 23–26].

The objective of the present study was to investigate the frequency of* IDH1*,* IDH2*, and* TP53 *mutations in Bulgarian patients with glioblastomas and their association with survival time and various clinical features in a search for prognostic factors.

## 2. Materials and Methods

### 2.1. Samples

Tumour tissues from 106 glioblastoma patients have been collected since 2005 in the Department of Neurosurgery, University Multiprofile Hospital for Active Treatment and Emergency Medicine (UMHATEM) “N.I.Pirogov,” Sofia, Bulgaria. All samples were diagnosed and independently histopathologically confirmed in the Department of Pathology, UMHATEM, by experienced neuropathologist according to the 2007 WHO classification system of CNS tumours [27]. GBMs were classified as primary when there was no clinical history of prior lower grade tumour and as secondary when patients had initially lower grade gliomas that progressed to GBM. The patients included 53 males and 53 females with a median age of 56 years old. This age was used to divide the cohort in two groups: young (less than 56 years old) versus old (more than 56 years old). In order to compare the gliomas that developed in one patient, we included tumour samples from two consecutive surgeries of 3 GBM patients and those from 3 surgical extractions in one patient initially diagnosed with astrocytoma WHO grade II, followed by GBM WHO grade IV. The study was approved by the Ethical Committee of Medical University of Sofia and informed consent was obtained from every patient.

### 2.2. DNA Extraction and PCR Amplification

DNA was extracted from 64 fresh-frozen and 47 FFPE tumour tissues using the QIAamp DNA Mini kit (Qiagen, Valencia, CA), according to the manufacturer's recommendations. The quantity of the DNA was assessed using NanoDrop 1000 spectrophotometer (Thermo Scientific, Wilmington, DE). For the detection of mutations, primers were designed to amplify exon 4 of the* IDH1*, exon 4 of the* IDH2*, and exons 5-8 of the* TP53* gene using Primer3 software (http://frodo.wi.mit.edu/primer3/) (Supplementary Table ST1 available online at http://dx.doi.org/10.1155/2014/654727). Polymerase chain reaction amplification was performed in a total volume of 10 *μ*L including 30 ng DNA, 0.5 *μ*M each primer, and 1xRedTaq PCR Master Mix (Sigma-Aldrich Co. LLC, St. Louis, MO, USA). The reaction mixture was subjected to initial denaturation at 95°C for 10 minutes, followed by 40–45 cycles consisting of denaturation at 95°C for 30 seconds, annealing at required temperature (Supplementary Table ST1) for 30 seconds, extension at 72°C for 40 seconds, and a final extension at 72°C for 10 minutes.

### 2.3. Direct Sequencing

A total of 1.5 *μ*L of each amplified product was subjected to ExoSap treatment followed by direct sequencing in both directions with the primers listed above using the BigDye Terminator v3.1 Sequencing Kit (Applied Biosystems, Foster City, CA, USA). The sequencing reaction was run on ABI 3130xl Genetic Analyzer (Applied Biosystems) according to the supplier's protocol and as described elsewhere [28]. The SeqScape Software v.2.5 (Applied Biosystems) was used for the interpretation of the sequence electropherograms.

### 2.4. Loss of Heterozygosity (LOH) Analysis

LOH was studied by fragment analysis of the microsatellite markers D15S996, D15S116, D15S202, and D15S127 in tumour and paired normal glial cells. Primer sequences for each marker were retrieved from NCBI's UniSTS database (http://www.ncbi.nlm.nih.gov/sites/entrez?db=unists) and each forward primer was labeled on the 5′ end with one of the two fluorescent dyes 6-FAM or HEX. PCR amplification was performed in one multiplex reaction with only 30 cycles of denaturing, annealing, and extension at 95°C for 30 s, 55°C for 30 s, and 72°C for 40 s, respectively. PCR products were separated on a 3130xl DNA Analyzer (Applied Biosystems). The GeneMapper Software v.4.0 (Applied Biosystems) was used for visualization of microsatellite markers.

### 2.5. Statistical Analysis

Statistical processing of the data was performed using Statistical Package for Social Sciences (SPSS) ver.17.0 (SPSS Inc., Chicago, IL, USA). Overall survival (OS) was calculated from the date of diagnosis until death or end of follow-up. Kaplan-Meier survival curves were plotted and the log-rank test was used to compare survival between groups. Also, univariate and multivariate Cox regression analyses were done to test the association of various factors with survival time. Six patients were excluded from the analysis because of insufficient clinical data.

## 3. Results

### 3.1. Genetic Aberrations in* IDH1*,* IDH2*, and* TP53* Genes

A total of 111 glioma samples from 106 patients were analyzed for* IDH1, IDH2*, and* TP53* mutations. Genetic aberrations in at least one of the genes were found in 46 samples from 42 patients (39.6%) (Supplementary Table ST2). Exon 4 of* IDH1* gene was mutated in 13 patients (12.3%). All* IDH1* aberrations were missense mutations at codon 132 leading to the substitution arginine to histidine (c.395G>A, R132H) (Table 1).

A sample with a homozygous R132H mutation in* IDH1* was found (Supplementary Figure SF1). Deletion of the second* IDH1* allele was suspected but could not be confirmed due to lack of normal tissue from the patient. Another novel aberration in homozygous state was detected in* IDH2*, c.386A>G, affecting codon 129. This mutation led to lysine-to-arginine replacement. Deletion of the second* IDH2* allele was confirmed by LOH analysis for the microsatellite markers D15S996, D15S116, D15S202, and D15S127 (Supplementary Figure SF2). LOH was observed in tumour DNA for three of the markers (D15S116, D15S202, and D15S127). D15S996 and D15S116 are located centromerically, while D15S202 and D15S127 are telomeric to IDH2, confirming LOH of at least 1.38 Mb including the entire* IDH2* gene. Further, three algorithms were employed to predict the impact of this substitution on protein activity. It was classified as “low confidence” with a score rate of 3.46 through SIFT (http://sift.jcvi.org/), as “benign” with a score rate of 0.270 using PolyPhen-2 (http://genetics.bwh.harvard.edu/pph2/), and as “not very reliable” using the SNPs&GO (http://snps-and-go.biocomp.unibo.it/snps-and-go/).

All samples were analyzed for mutations in hot-spot exons 5 to 8 of* TP53*, encoding its DNA binding domain.* TP53* mutations were detected in 37 patients (34.9%). Four patients carried two simultaneous mutations in* TP53* gene. Of the 41 aberrations found, 9 (21.9%) were in exon 5, 4 (9.8%) in exon 6, 12 (29.3%) in exon 7, and 16 (39%) in exon 8. The most frequent* TP53* mutations were c.773A>T (E258V) in exon 7 and c.817C>T (R273C) in exon 8, each detected in 5 patients (Table 1). Nine of the patients with* IDH1/IDH2* mutations (64%) carried also mutated* TP53* (Supplementary Table ST2).

Comparing the samples from two consecutive operations of 3 patients we found the same mutation status: two patients carried the same* TP53* mutation and one patient had no mutations. However, there was a difference in the patient with samples from three surgeries. The first operation was in 2005 with diagnosis astrocytoma WHO grade II (sample 16, Supplementary Table ST2). A mutation in* IDH1* (R132H) and in exon 7 of* TP53* (del 759_61) was found. The sample from the second surgery in 2009 (sample 52) revealed the same two aberrations although the tumour had developed in glioblastoma. In 2010 the third operation was performed but the tumour (sample 41) did not carry the deletion in exon 7 of* TP53*. As all the resections were subtotal, it might be speculated that during the second surgery the part of the tumour carrying* TP53* mutated clone was removed, but the cells with* IDH1* mutation were not and they formed the third glioma.

Except for the above mutations one synonymous change in* IDH1* and 2 aberrations in intron 6 of* TP53* were found. The SNP in* IDH1*, c. 315G>T, G105G (rs11554137) was located in exon 4 in the same region where the R132 mutation was found. The SNP was detected in 9 patients, one of them harbouring also the mutation R132H. The aberrations found in intron 6 of* TP53* were rs17884607 (g.7578129T>C) and rs34949160 (g.7578146C>T).

### 3.2. Association of Mutation Status with Clinical Features

Table 2 shows the distribution of mutations in* IDH1/IDH2* and* TP53* among patients according to their clinical characteristics. Mutations were predominantly found in young patients (age ≤ 56 years) with higher KPS (>70). This trend was more pronounced in the group with* IDH1/IDH2* mutations than in the one with* TP53* mutations. In confirmation of the previous publications* IDH1/IDH2* mutations were more frequent in secondary than in primary GBM. Also, median OS was longer in patients harbouring aberrations, 30.9 months for* IDH1/IDH2* mutated and 9.1 months for* TP53* mutated, compared to 6.2 months for the group without mutations.

Clinical data of the patients with homozygous* IDH1*/*IDH2* mutations were analyzed. The patient with homozygous* IDH1* mutation was 53 years old and had OS of 34.6 months; the tumour was a primary GBM and surgery was followed by both chemo- and radiotherapy. The clinical features seemed similar to the others carrying heterozygous* IDH1* mutations. The patient with the novel mutation in* IDH2* gene had long OS (30.3 months), as expected for a secondary GBM, although he was 74 years old. Because of the long OS this case was added to those with mutations in* IDH1* for further analysis.

**Figure 1:**
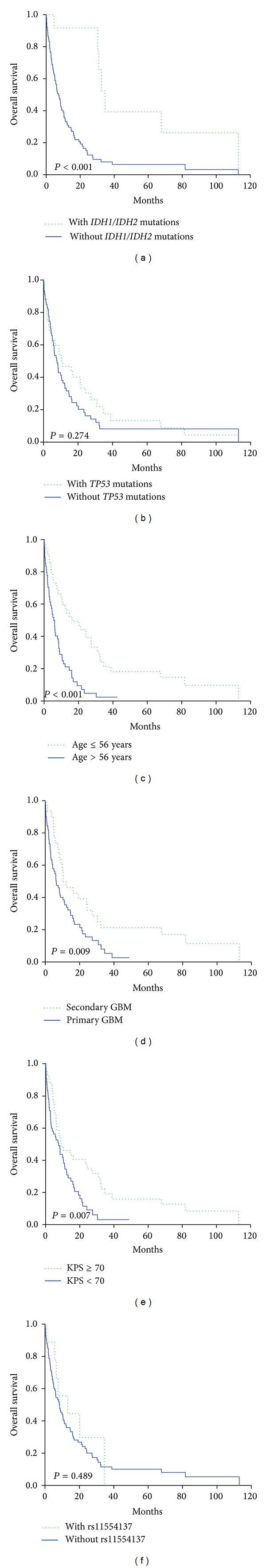
Kaplan-Meier plots of glioma patients showing the association of the following factors with overall survival: (a)* IDH1/IDH2* mutations, (b)* TP53* mutations, (c) age, (d) primary versus secondary GBM, (e) KPS, and (f)* IDH1* rs11554137.

Univariate analysis using log-rank test was used to evaluate the prognostic value of* IDH1/IDH2* mutations; the patients carrying mutations had better OS (*P* = 0.001) (Figure 1(a)). Although genetic aberrations in* IDH1/IDH2* have been correlated with those in* TP53* [14], no significant difference in survival was found between mutated and nonmutated* TP53* groups (median survival of 9.1 versus 7.6 months) (Figure 1(b)). Other factors with prognostic value were also examined. The younger patients showed longer OS (*P* < 0.001) (Figure 1(c)). Further, the secondary GBM was the favourable type (*P* = 0.001) (Figure 1(d)). KPS over 70 predicted longer OS as well (Figure 1(e)). The impact of the* IDH1* polymorphism was also analysed, but no association with OS was found (*P* = 0.454) (Figure 1(f)).

Cox proportional hazards model was used to calculate the hazard ratios for each of the examined factors (Table 3). Univariate Cox regression demonstrated significant association of OS with the age (per year), type of GBM (primary versus secondary), and* IDH1/IDH2* mutation status. In the multivariate analysis only age and* IDH1/IDH2* mutation status showed statistical significance as independent prognostic factors. While age was a predictor of poor survival,* IDH1/IDH2* mutations were predicting longer OS (Table 3).

**Figure 2:**
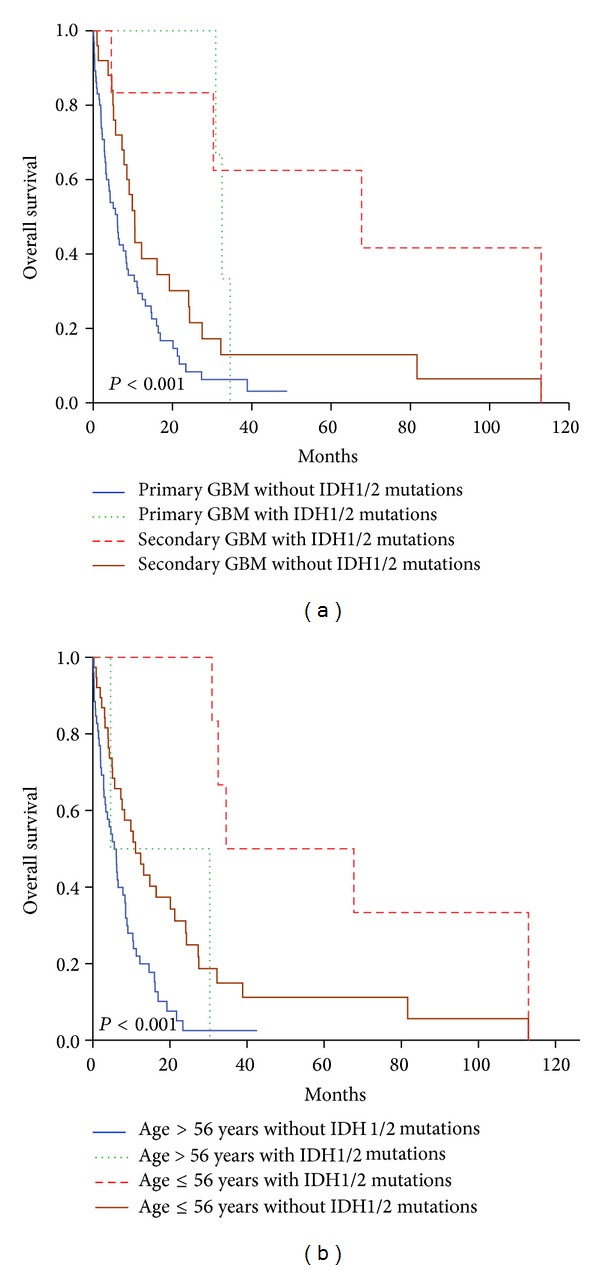
Kaplan-Meier plots of overall survival in groups divided by two factors:* IDH1/IDH2* mutation status and (a) primary versus secondary GBM and (b) age.

We investigated further the impact of* IDH1/IDH2* mutations on survival in groups stratified by the type of GBM (Figure 2). When the patients were divided into primary and secondary GBM the most favourable group included patients with secondary GBM and* IDH1/IDH2* mutations (median OS: 68.6 months) while the worst was primary GBM with no* IDH1/IDH2* aberrations (median OS: 5.5 months). It was also observed that the group with secondary GBMs without IDH1/IDH2 mutations had a worse overall survival than the group with primary GBM but with mutated IDH1/IDH2 (Figure 2(a)).

Coevaluation of age and* IDH1/IDH2* mutation status showed that both factors contribute to prognosis (Figure 2(b)). Younger patients with mutated* IDH1*/*IDH2* had the most favourable clinical course while those over 56 and carrying wild-type* IDH *genes were with the worst prognosis (*P* < 0.001).

## 4. Discussion

In the present study we focused on the investigation of prognostic markers for patients with glioblastomas, especially genetic aberrations in* IDH1, IDH2,* and* TP53* genes. Mutations in R132 of IDH1 were detected in only 12.3% of patients, probably due to the high number of primary GBM (93 cases) in the study (Table 2). The mutations were more frequent in secondary glioblastomas (30.7%). This frequency is still much lower than reported [12–14, 16, 17, 20, 29] and this could be due to the small number of secondary GBMs in our study group. Also, this was the first investigation in Bulgaria evaluating the frequency of the IDH1/IDH2 mutations in GBM patients and there was no data, which could be used as a reference for Bulgarian population frequencies. Furthermore, we classified tumours as primary GBM only on the basis of lack of previously detected glial tumour, and thus a few secondary tumours might have been missed.

In accordance with previous publications, 64% of* IDH1/IDH2* mutated tumours harboured also a mutation in* TP53* [7, 14, 23].

Normally both cytosolic IDH1 and mitochondrial IDH2 exist as homodimers [30]. Analysis of the effect of heterozygous* IDH1* mutations on glioma cells has shown that mutant IDH1 affects the ability of the enzyme to reduce *α*-ketoglutarate to 2-hydroxyglutarate (2HG) [31]. The 2HG-producing IDH mutants can prevent the histone demethylation required for the differentiation of lineage-specific progenitor cells. In glioma patients* IDH* mutations have been associated with a gene expression profile enriched for genes expressed in neural progenitor cells, which were correlated with increased histone methylation [32]. It has recently been shown that glioblastomas with the CpG island methylator phenotype (CIMP) are associated with the proneural subgroup of tumours and are driven by* IDH1* mutation [33, 34].

In the present study we found a homozygous mutation in codon 132 (R132H) in a GBM patient. Deletion of the normal allele was suspected, as both the mutation and the polymorphism in* IDH1* gene were in a homozygous state. Recently homozygous* IDH1* aberrations in gliomas were reported for the first time in 2 patients with secondary GBM [35] and in a patient with astrocytoma [36]. Previously, homozygous* IDH1* mutations have been detected in leukemia [37] and thyroid cancer patients [38]. Jin et al. have reported lower 2HG levels in the cells lacking wild-type* IDH1* allele compared to the ones with heterozygous mutations which might hamper application of 2HG-level modulators as a potential therapeutic strategy for IDH1-mutated tumours [35]. In line with the current finding that 2-HG production, but not dominant negative function, is conferred by glioma-derived NADP+-dependent* IDH* mutations [39], coupled with their role to block the histone demethylation and associate with CIMP [32], we could speculate that in the homozygous R132H carrier the extent of methylation will be greatly increased compared to the heterozygote.

The role of* IDH2* mutations alone is still unclear because of the rare occurrence in gliomas: 1-2% [28]. It is more common in acute myeloid leukemia (AML), about 15%, where hot spots for mutations are codons 140 and 172 [40]. Although the examined cohort included 106 patients we did not find any mutations in the reported codons but found a novel aberration in codon 129, c.386A>G, K129R. Both lysine and arginine are basic amino acids, but the change affected a part of a conservative domain in IDH2. Also, the second allele was lost. However, analyses of the variant with SIFT, PolyPhen, and SNPs&GO showed that most likely the change is not pathogenic.

Several previous studies have demonstrated the important role of* IDH1*/*IDH2* mutations for determining the prognosis of glioblastoma patients [6, 17, 20]. Our group of patients was with short median overall survival of 7.7 months, which was lower than the most published data [41, 42]. One of the reasons for this could be the predominant number of primary GBM in our cohort. Ohgaki and coauthors reported lower median survival in primary GBM (4.7 months) than in secondary GBM (7.8 months) [26], and even though only half of their patients underwent surgical treatment, while all of our patients had surgery, their data were close to ours; median OS was 6.2 and 25.8 months for primary and secondary GBMs, respectively [26]. Another reason could be the changes in radio- and/or chemotherapy strategy through the years. Bleeker et al. published data of tumours, which have been under treatment before 2006 and were with a similar median OS to ours, 8.7 months [43]. However, we confirmed that patients with IDH1/2 mutations had much longer OS than those with no mutation (Figure 1(a)).

In addition,* IDH1/IDH2* mutations were found predominantly in younger patients. Although 64% of* IDH1/IDH2* mutated tumours carried also* TP53* mutations, an investigation over OS of groups with and without* TP53* mutations did not show any significant difference. Moreover, no favourable prognosis was found in the group of patients with affected codon 248 or 273 of the* TP53* in accordance with previous reports [25].

The impact of the polymorphism in* IDH1* (rs11554137) on survival has been investigated in AML and thyroid carcinoma. Although the SNP has not been significant for thyroid carcinogenesis [44], in AML patients it has been associated with poor prognosis and higher expression of* IDH1* mRNA [45]. This aberration has been suspected to alter IDH1 activity by changing RNA stability, folding, and splicing [45]. However, in our study rs11554137 did not show association with OS of glioma patients (Table 3).

Multivariate regression analysis determined age and* IDH1/IDH2* mutation status as independent prognostic factors (Table 3). According to Hartmann et al.,* IDH1* mutations are better predictors of survival than histopathology [46]. Although we studied only GBM cases, our results showed that IDH1/IDH2 mutations were more powerful prognostic factors than primary/secondary GBM classification; we observed better OS in primary GBM with mutated* IDH1/IDH2* compared to the secondary GBM with no mutation (Figure 2(a)). It is likely that* IDH1* mutations are favourable for prognosis independently of the GBM type. On the other hand, coevaluation of age and* IDH1/IDH2* mutation status revealed predictive value of* IDH1/IDH2* only in the group of patients below 56 years of age(Figure 2(b)). This might be a result of the small number of mutations in older patients (Table 2).

## 5. Conclusions

Even though in gliomas mutations in* IDH1* and* IDH2 *genes are usually heterozygous, homozygous ones also exist.* IDH1/IDH2* aberrations, but not the ones in* TP53*, together with age might be applied in clinical practice for prediction of survival in patients with glioblastomas.

## Supplementary Material

Supplementary Table 1: Sequences of the primers and optimal annealing temperatures (Tan) for PCR amplification.
Supplementary Figure 1: The mutation in IDH1 gene c.395G>A (R132H) (a) Homozygous wild type, genotype GG; (b) Heterozygous substitution, genotype GA; (c) Homozygous mutation, genotype AA.
Supplementary Figure 2: LOH analysis for the microsatellite markers flanking the IDH2 locus on 15q.

## Figures and Tables

**Table 1:** Mutationsin *IDH1*,* IDH2*, and *TP53* genes that were detected in 106 brain tumour patients.

Gene	Exon	Nucleotide change (amino acid change)		Number of patients with mutations (which are homozygous)	% mutations among all mutations in the gene
*IDH1 *	Exon 4	c.395G>A	(R132H)	13 (1)	100 (7.7)
*IDH2 *	Exon 4	c.386A>G	(K129R)	1 (1)	100

*TP53 *	Exon 5	c.427G>A	(V143M)	1	2.4
		c.455C>T	(P152L)	1	2.4
		c.473G>A	(R158H)	1 (1)	2.4 (2.4)
		c.495G>C	(Q165H)	1	2.4
		c.523C>A	(R175S)	1	2.4
		c.524G>A	(R175H)	3	7.3
		c.535C>A	(H179N)	1	2.4
	Exon 6	c.584T>C	(I195T)	1	2.4
		c.632C>T	(T211I)	1	2.4
		c.643A>G	(S215G)	1	2.4
		c.659A>G	(Y220C)	1	2.4
	Exon 7	c.725G>A	(C242Y)	1	2.4
		c.733G>A	(G245S)	1	2.4
		c.742C>T	(R248W)	2	4.9
		c.773A>T	(E258V)	5	12.2
		c.775G>C	(D259H)	1	2.4
		del 716_21	(NA)	1	2.4
		del 759_61	(NA)	1	2.4
	Exon 8	c.799C>T	(R267W)	1	2.4
		c.806G>T	(S269I)	1	2.4
		c.817C>T	(R273C)	5 (3)	12.2 (7.3)
		c.821T>C	(V274A)	1	2.4
		c.841G>C	(D281H)	3	7.3
		c.844C>T	(R282W)	1	2.4
		c.847C>T	(R283C)	1	2.4
		c.850A>T	(T284S)	1	2.4
		c.853G>A	(E285K)	1	2.4
		c.857A>T	(E286V)	1	2.4

**Table 2:** Clinical features of the patients with mutations in *IDH1/IDH2* and *TP53* genes.

Clinical characteristics	*N* (*n* = 106)	*IDH1/IDH2* mutated (*n* = 14)	*TP53* mutated (*n* = 37)
Gender			
Male	53	8 (15.1%)	20 (37.7%)
Female	53	6 (11.3%)	17 (32.1%)
Age			
<56 years old	51	12 (23.5%)	22 (43.1%)
≥56 years old	55	2 (0.36%)	15 (27.3%)
Karnofsky Scale^a^			
KPS ≤70	56	3 (0.5%)	15 (26.8%)
KPS >70	45	10 (22.2%)	21 (46.6%)
Resection^a^			
Subtotal or partial	54	10 (18.5%)	21 (38.8%)
Total	50	4 (0.8%)	16 (32%)
Glioblastoma type			
Primary	93	10 (10.7%)	29 (31.2%)
Secondary	13	4 (30.7%)	8 (61.5%)
Overall survival^a^ (months)			
Median	7.7	30.9	9.1

^a^Missing data for several patients.

*N*: number of patients.

**Table 3:** Univariate and multivariate Cox analyses of the association between the features of the patients and overall survival.

	Univariate Cox regression			Multivariate Cox regression		
	HR	95% CI	*P *	HR	95% CI	*P *
Age (per year)	1.048	1.029–1.067	<0.001	1.039	1.018–1.061	<0.001
Gender (female versus male)	0.884	0.575–1.361	0.576	∗	∗	∗
Secondary versus primary GBM	0.271	0.123–0.597	0.001	0.487	0.215–1.106	0.086
Resection (total versus others)	0.854	0.559–1.303	0.463	∗	∗	∗
KPS (per 10 points)	0.990	0.979–1.001	0.083	∗	∗	∗
*IDH1/IDH2* (mutated versus nonmutated)	0.274	0.124–0.604	0.001	0.237	0.157–0.791	0.011
*IDH1* SNP (SNP versus no SNP)	0.745	0.343–1.617	0.456	∗	∗	∗
*TP53* (mutated versus nonmutated)	0.764	0.489–1.196	0.240	∗	∗	∗

*Not included in multivariate analysis.

HR: hazard ratio; CI: confidence interval; *P*: *P* value; *IDH1* SNP refers to rs11554137.
