# Supplementary material for: IDH1/IDH2 but Not TP53 Mutations Predict Prognosis in Bulgarian Glioblastoma Patients
Source: Biomed Res Int. 2014 Apr 24;2014:654727. doi: 10.1155/2014/654727 (PMC4017788; doi:10.1155/2014/654727)
Supplement: Supplementary file 1 — Supplementary Table 1: Sequences of the primers and optimal annealing temperatures (Tan) for PCR amplification. Supplementary Figure 1: The mutation in IDH1 gene c.395G>A (R132H) (a) Homozygous wild type, genotype GG; (b) Heterozygous substitution, genotype GA; (c) Homozygous mutation, genotype AA. Supplementary Figure 2: LOH analysis for the microsatellite markers flanking the IDH2 locus on 15q. [file 654727.f1.doc]

***IDH1/ IDH2*, but not *TP53* mutations predict prognosis in Bulgarian glioblastoma patients**


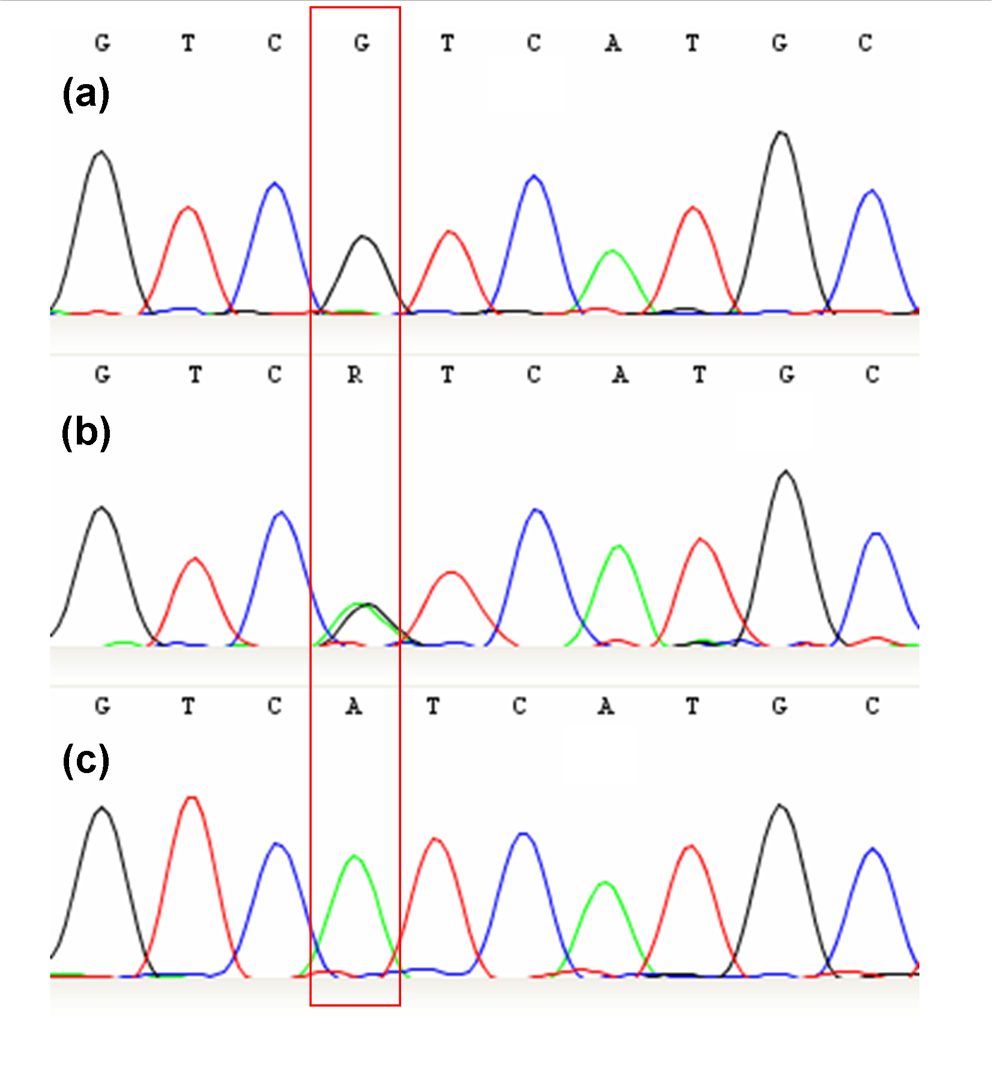


**Supplementary Fig. SF1** The mutation in *IDH1* gene c.395G>A (R132H): (**a**) Homozygous wild type, genotype GG; (**b**) Heterozygous substitution, genotype GA; (**c**) Homozygous mutation, genotype AA.


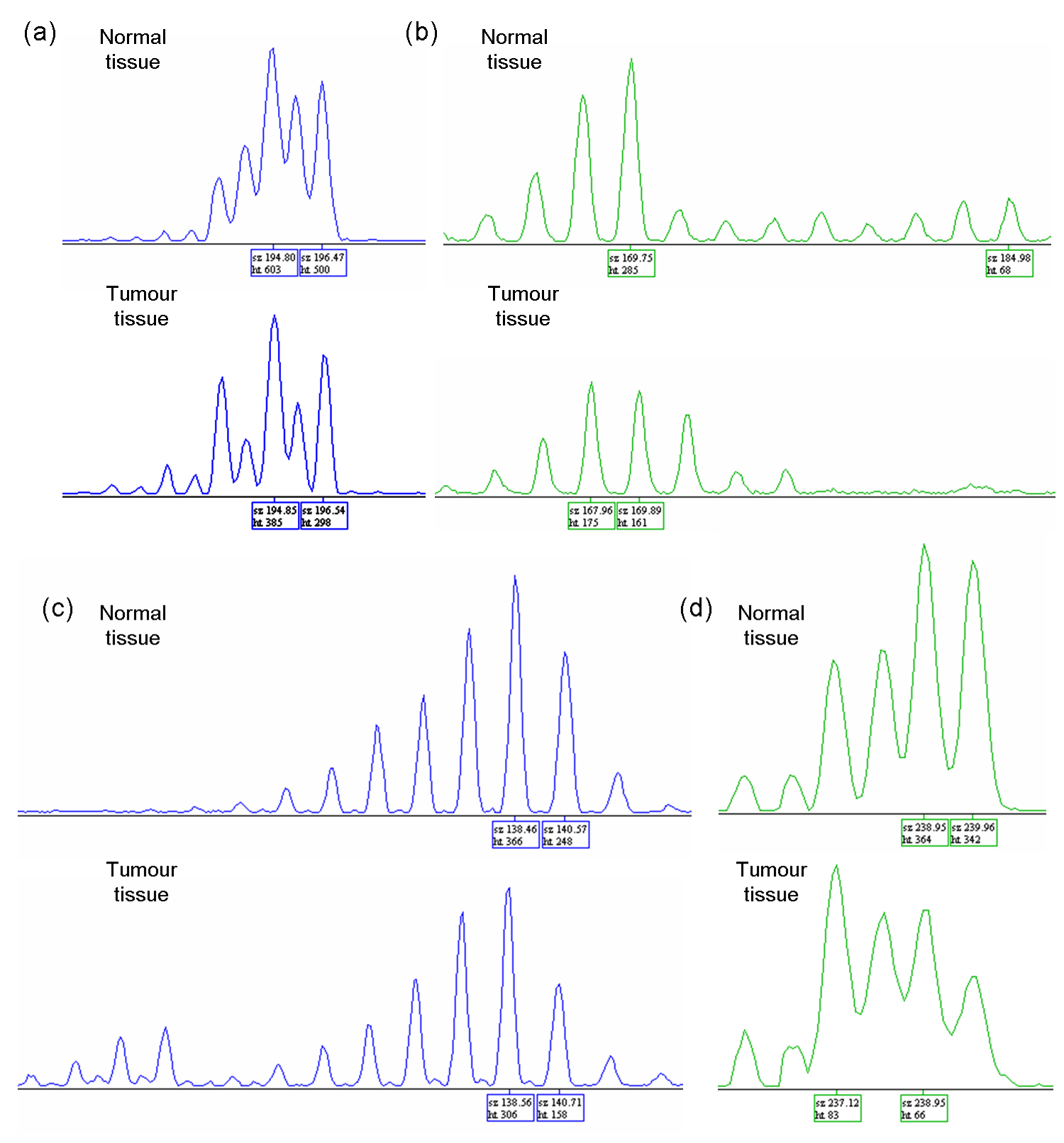


**Supplementary Fig. SF2** LOH analysis for the microsatellite markers flanking the IDH2 locus on 15q:

(**a**) D15S996; (**b**)D15S116; (**c**) D15S202; (**d**) D15S127

**Supplementary Table ST1** Sequences of the primers and optimal annealing temperatures (T_an_) for PCR amplification

| **Gene** | **Primer sequence** | **Amplicon size**  **(bр)** | **T_an_ (^o^C)** |
| --- | --- | --- | --- |
| *IDH1* | F 5`- AGCTCTATATGCCATCACTGC -3` | 496 | 62°C |
|  | R 5`- TTCATACCTTGCTTAATGGGTGT -3` |  |  |
| *IDH2* | F 5`- ATTCTGGTTGAAAGATGGCG -3` | 313 | 62°C |
|  | R 5`- TGTGGCCTTGTACTGCAGAG -3` |  |  |
| *TP53_ex.5* | F 5`- CTTTGCTGCCGTCTTCCAGTT -3` | 334 | 66°C |
|  | R 5`- TCAGTGAGGAATCAGAGGCCT -3` |  |  |
| *TP53_ex.6* | F 5`- CTGGGGCTGGAGAGACGACA -3` | 257 | 72°C |
|  | R 5`- CTGCTCACCTGGAGGGCCACT -3` |  |  |
| *TP53_ex.7* | F 5`- CCCCTGCTTGCCACAGGT -3` | 240 | 72°C |
|  | R 5`- GGTCAGAGGCAAGCAGAGG -3` |  |  |
| *TP53_ex.8* | F 5`- ATGGGACAGGTAGGACCTGA -3` | 259 | 61°C |
|  | R 5`- GTGAATCTGAGGCATAACTGC -3` |  |  |

**Supplementary Table ST2** Genetic aberrations found in 111 brain tumour samples

| **Sample No.** | **Diagnosis/**  **WHO grade** | **Primary/**  **Secondary** | ***IDH1***  **aberration** | ***IDH1***  **rs 11554137** | ***IDH2***  **aberration** | ***TP53***  **aberration** | | | | |
| --- | --- | --- | --- | --- | --- | --- | --- | --- | --- | --- |
| 2 | glioblastoma  IV | primary | - | - | - | **c.725 G>A C242Y** | | | | |
| 4 | glioblastoma  IV | primary | - | - | - | **c.853G>A E285K** | | | | |
| 6 | glioblastoma  IV | primary | - | - | - | **c.535C>A H179N** | | | | |
| 9 | glioblastoma  IV | primary | - | - | - | - | | | | |
| 10 | glioblastoma  IV | primary | - | - | - | - | | | | |
| 12 | glioblastoma  IV | primary | - | - | - | - | | | | |
| 13 | glioblastoma  IV | primary | - | - | - | **c.524G>A R175H** | | | | |
| 14 | glioblastoma  IV | primary | **c.395G>A R132H** | - | - | **c.523C>A R175S** | | | | |
| 15 | glioblastoma  IV | primary | - | - | - | - | | | | |
| 16**^a^** | astrocytoma  II | - | **c.395G>A R132H** | - | - | **del 759_61** | | | | |
| 18 | glioblastoma  IV | primary | - | - | - | - | | | | |
| 19 | glioblastoma  IV | secondary | - | - | - | - | | | | |
| 21 | glioblastoma  IV | primary | **c.395G>A R132H** | - | - | **c.817C>T R273C** | | | | |
| 22 | glioblastoma  IV | primary | - | - | - | - | | | | |
| 23 | glioblastoma  IV | primary | - | **C>T** | - | - | | | | |
| 25 | glioblastoma  IV | primary | - | - | - | - | | | | |
| 27 | glioblastoma  IV | primary | - | - | - | - | | | | |
| 29 | glioblastoma  IV | primary | - | **C>T** | - | - | | | | |
| 31 | glioblastoma  IV | primary | **c.395G>A R132H** | - | - | - | | | | |
| 32 | glioblastoma  IV | primary | - | **C>T** | - | **c.524G>A R175H** | | | | |
| 33 | glioblastoma  IV | primary | - | - | - | **del 716_21** | | | | |
| 35 | glioblastoma  IV | primary | - | - | - | - | | | | |
| 36 | glioblastoma  IV | primary | - | - | - | - | | | | |
| 37 | glioblastoma  IV | primary | - | **C>T** | - | **c.455C>T P152L** | | | | |
| 38 | glioblastoma  IV | secondary | - | - | - | **c.584T>C I195T** | | | | |
| 40 | glioblastoma  IV | primary | - | - | - | - | | | | |
| 41**^a^** | glioblastoma  IV | secondary | **c.395G>A R132H** | - | - | - | | | | |
| 42 | glioblastoma  IV | primary | **c.395G>A R132H** | - | - | **c.817C>T** **^е^ R273C** | | | | |
| 44 | glioblastoma  IV | primary | - | - | - | - | | | | |
| 45 | glioblastoma  IV | primary | - | - | - | **rs34949160** | | | | |
| 46 | glioblastoma  IV | primary | - | - | - | - | | | | |
| 47 | glioblastoma  IV | primary | - | - | - | **c.773A>T E258V** | | | | |
| 48**^b^** | glioblastoma  IV | primary | - | - | - | **c.659A>G Y220C** | | | | |
| 49 | glioblastoma  IV | primary | - | **C>T** | - | - | | | | |
| 50**^c^** | glioblastoma  IV | primary | - | - | - | - | | | | |
| 51**^d^** | glioblastoma  IV | primary | - | - | - | **c.806G>T S269I** | | | | |
| 52**^a^** | glioblastoma  IV | secondary | **c.395G>A R132H** | - | - | **del 759_61** | | | | |
| 53 | glioblastoma  IV | secondary | - | - | - | **c.742C>T R248W** | | | **c.799C>T R267W** | |
| 55 | glioblastoma  IV | primary | **c.395G>A R132H** | - | - | **c.775G>C D259H** | | | | |
| 56 | glioblastoma  IV | primary | - | - | - | **c.841G>C D281H** | | | | |
| 57 | glioblastoma  IV | primary | - | - | - | - | | | | |
| 59 | glioblastoma  IV | primary | - | - | - | **c.844C>T R282W** | | | | |
| 60 | glioblastoma  IV | primary | - | - | - | - | | | | |
| 61 | glioblastoma  IV | primary | - | - | - | - | | | | |
| 62 | glioblastoma  IV | primary | **c.395G>A** **^е^ R132H** | **C>T** | - | **c.742C>T R248W** | | | | |
| 63**^d^** | glioblastoma  IV | primary | - | - | - | **c.806G>T S269I** | | | | |
| 65**^b^** | glioblastoma  IV | primary | - | - | - | **c.659A>G Y220C** | | | | |
| 66 | glioblastoma  IV | secondary | - | - | - | - | | | | |
| 67**^c^** | glioblastoma  IV | primary | - | - | - | - | | | | |
| 68 | glioblastoma  IV | secondary | - | - | - | **c.817C>T** **^е^ R273C** | | | | |
| 69 | glioblastoma  IV | primary | - | - | - | - | | | | |
| 70 | glioblastoma  IV | secondary | - | - | - | - | | | | |
| 71 | glioblastoma  IV | primary | **c.395G>A R132H** | - | - | **c.841G>C D281H** | | | | |
| 72 | glioblastoma  IV | primary | - | - | - | - | | | | |
| 73 | glioblastoma  IV | primary | - | - | - | - | | | | |
| 74 | glioblastoma  IV | primary | - | - | - | - | | | | |
| 76 | glioblastoma  IV | primary | - | - | - | - | | | | |
| 77 | glioblastoma  IV | primary | - | - | - | - | | | | |
| 78 | glioblastoma  IV | primary | - | - | - | - | | | | |
| 79 | glioblastoma  IV | primary | - | - | - | - | | | | |
| 80 | glioblastoma  IV | primary | - | - | - | **c.773A>T E258V** | | | | |
| 81 | glioblastoma  IV | primary | - | - | - | **c.857A>T E286V** | | | | |
| 82 | glioblastoma  IV | primary | - | - | - | - | | | | |
| 83 | glioblastoma  IV | primary | - | - | - | - | | | | |
| 84 | glioblastoma  IV | primary | - | - | - | - | | | | |
| 85 | glioblastoma  IV | primary | - | - | - | **c.632C>T T211I** | **c.773A>T E258V** | | | **rs34949160** |
| 86 | glioblastoma  IV | primary | - | - | - | **c.773A>T E258V** | | | **c.847C>T R283C** | |
| 89 | glioblastoma  IV | primary | - | - | - | **c.773A>T E258V** | | | | |
| 93 | glioblastoma  IV | primary | - | - | - | - | | | | |
| 95 | glioblastoma  IV | primary | - | - | - | **c.841G>C D281H** | | | | |
| 97 | glioblastoma  IV | primary | - | - | - | - | | | | |
| 102 | glioblastoma  IV | primary | - | - | **c.386A>G ^е^ K129R** | - | | | | |
| 103 | glioblastoma  IV | primary | - | - | - | - | | | | |
| 104 | glioblastoma  IV | secondary | - | - | - | **c.850A>T T284S** | | | | |
| 105 | glioblastoma  IV | secondary | - | - | - | **c.817C>T** **^е^ R273C** | | | | |
| 106 | glioblastoma  IV | primary | - | - | - | - | | | | |
| 109 | glioblastoma  IV | primary | - | - | - | **c.427G>A V143M** | | | | |
| 112 | glioblastoma  IV | primary | - | - | - | - | | | | |
| 117 | glioblastoma  IV | primary | - | - | - | - | | | | |
| 118 | glioblastoma  IV | primary | - | - | - | - | | | | |
| 128 | glioblastoma  IV | primary | - | - | - | - | | | | |
| 134 | glioblastoma  IV | primary | - | - | - | - | | | | |
| 135 | glioblastoma  IV | primary | - | - | - | - | | | | |
| 137 | glioblastoma  IV | primary | - | **C>T** | - | - | | | | |
| 138 | glioblastoma  IV | primary | - | - | - | - | | | | |
| 139 | glioblastoma  IV | primary | - | - | - | - | | | | |
| 140 | glioblastoma  IV | primary | - | - | - | - | | | | |
| 141 | glioblastoma  IV | primary | - | - | - | **rs17884607** | | | | |
| 142 | glioblastoma  IV | primary | - | - | - | - | | | | |
| 144 | glioblastoma  IV | primary | - | - | - | - | | | | |
| 145 | glioblastoma  IV | primary | - | - | - | - | | | | |
| 146 | glioblastoma  IV | primary | - | - | - | - | | | | |
| 147 | glioblastoma  IV | primary | - | - | - | - | | | | |
| 148 | glioblastoma  IV | primary | - | - | - | - | | | | |
| 149 | glioblastoma  IV | secondary | - | - | - | **c.524G>A R175H** | | | | |
| 150 | glioblastoma  IV | primary | - | **C>T** | - | - | | | | |
| 159 | glioblastoma  IV | secondary | **c.395G>A R132H** | - | - | - | | | | |
| 160 | glioblastoma  IV | primary | - | - | - | - | | | | |
| 161 | glioblastoma  IV | primary | **c.395G>A R132H** | - | - | **c.817C>T**  **R273C** | | | | |
| 162 | glioblastoma  IV | primary | - | - | - | - | | | | |
| 163 | glioblastoma  IV | primary | - | - | - | - | | | | |
| 164 | glioblastoma  IV | primary | - | - | - | **c.821T>C**  **V274A** | | | | |
| 166 | glioblastoma  IV | secondary | **c.395G>A R132H** | - | - | **c.473G>A ^е^**  **R158H** | | | | |
| 167 | glioblastoma  IV | secondary | **c.395G>A R132H** | - | - | - | | | | |
| 168 | glioblastoma  IV | primary | - | - | - | - | | | | |
| 170 | glioblastoma  IV | primary | - | - | - | - | | | | |
| 172 | glioblastoma  IV | primary | - | - | - | - | | | | |
| 174 | glioblastoma  IV | primary | - | **C>T** | - | - | | | | |
| 176 | glioblastoma  IV | primary | - | - | - | - | | | | |
| 177 | glioblastoma  IV | primary | **c.395G>A R132H** | - | - | - | | | | |
| 178 | glioblastoma  IV | primary | - | - | - | **c.495G>C**  **Q165H** | | **c.643A>G**  **S215G** | | |

**^a, b, c, d^** - the patients with more than one sample included in the study. Each sample was examined as an independent sample.

**^е^** - homozygous mutation
